# DM‐induced Hypermethylation of IR and IGF1R attenuates mast cell activation and airway responsiveness in rats

**DOI:** 10.1111/jcmm.16059

**Published:** 2020-11-03

**Authors:** Dan Fu, Hailu Zhao, Liang He, Huafeng Feng

**Affiliations:** ^1^ Department of Endocrinology Endocrinology Research Center Xiangya Hospital of Central South University Changsha China; ^2^ Diabetic Systems Center Guangxi Key Laboratory of Excellence Guilin Medical University Guilin China; ^3^ Department of Anesthesiology Affiliated Hospital of Guilin Medical University Guilin China

**Keywords:** airway responsiveness, asthma, diabetes mellitus, insulin receptor, insulin‐like growth factor 1 receptor, methylation

## Abstract

Diabetes has been reported to modulate the airway smooth muscle reactivity and lead to attenuation of allergic inflammatory response in the lungs. In this study, we aimed to study the effect of insulin on cell activation and airway responsiveness in patients with diabetes mellitus (DM). The airway contraction in rat model groups including a non‐DM group, a non‐DM+INDUCTION group, a DM+INDUCTION group and a DM+INDUCTION+INSULIN group was measured to observe the effect of insulin on airway responsiveness. Radioenzymatic assay was conducted to measure the levels of histamine, and ELISA assay was conducted to measure bronchial levels of interleukin (IL)‐1b, tumour necrosis factor (TNF)‐a, cytokine‐induced neutrophil chemoattractant (CINC)‐1, P‐selectin and β‐hexosaminidase. The tension in the main and intrapulmonary bronchi of DM+INDUCTION rats was lower than that of the non‐DM+INDUCTION rats, whereas the treatment of insulin partly restored the normal airway responsiveness to OA in DM rats. The release of histamine was remarkably suppressed in DM+INDUCTION rats but was recovered by the insulin treatment. Also, OA significantly increased the levels of IL‐1b, TNF‐a, CINC‐1 and P‐selectin in non‐DM rats, whereas insulin treatment in DM+INDUCTION rats partly restored the normal levels of IL‐1b, TNF‐a, CINC‐1 and P‐selectin in DM rats. Moreover, the expression of IR and IGF1R was evidently suppressed in DM rats, with the methylation of both IR and IGF1R promoters was aggravated in DM rats. Therefore, we demonstrated that DM‐induced hypermethylation inhibited mast cell activation and airway responsiveness, which could be reversed by insulin treatment.

## 1 INTRODUCTION

Asthma is a persistent condition primarily defined by coughing, episodic wheeze and shortness of breath caused by respiratory tract hyper‐responsiveness and inflammation. Asthma is among the most typical and persistent lung illness worldwide, impacting roughly 20 million adults in the United States.^1^, ^2^, ^3^ Mast cells are identified as cells associated with allergic problems such as asthma, and mast cell activation might cause the secretion of many cytokines linked to asthma, such as CXCL8, IL‐13 and IL‐5.^4^, ^5^, ^6^

Diabetes is actually a collection of metabolic illnesses featured with hyperglycaemia arising from the dysfunction in insulin release and activity. The persistent hyperglycaemia of diabetic patients can cause lasting damages of various body organs, specifically the eyes, nerves and the cardiovascular system. Numerous pathogenic processes were shown to be associated with the progression of diabetes mellitus, ranging from autoimmune damages of pancreatic cells to insulin resistance. The link between asthma and type I diabetes has been controversial. There are some studies presenting a much higher likelihood of asthma in individuals with type I diabetes.^7^, ^8^ Nonetheless, others present that the issues of hypersensitivity, such as asthma, are less likely in individuals with type I diabetes.^8^, ^9^

It was actually revealed that gestational diabetes mellitus (GDM) is related to several small changes in the methylation of IR DNA. It was also assumed that various other regulatory or epigenetic mechanisms are impacted in GDM, resulting in down‐regulated IR.^10^ Moreover, IGF1R was also shown to become methylated in DM, thus resulting in a decreased level of IGF1R expression in DM.^11^

Diabetes has been reported to modulate the airway smooth muscle reactivity and lead to attenuation of allergic inflammatory response in the lungs.^12^, ^13^ Insulin, which modulates the production/release of TNF‐a, IL‐1b and P‐selectin in diabetic rats, was also reported to affect pulmonary function and respiratory symptoms in patients with diabetes.^2^, ^14^ Moreover, it has been suggested that the activation of insulin signalling, including both IR and IGF1R signalling pathways, might modulate mast cell degranulation and activation.^15^ Therefore, it can be hypothesized that the hypermethylation of IR induced by DM could effect upon mast cell activation. And insulin could influence airway responsiveness in rats and expression of other cell cytokines in the respiratory system in DM rats. In this study, we studied the effect of insulin on airway responsiveness and the levels of TNF‐a, IL‐1b, CINC‐1 and P‐selectin in the respiratory system in DM rats. Also, the methylation status of IR/IGF1R promoters and the expression of IR/IGF1R were measured in vitro and in vivo to validate the effect of insulin.

## 2 MATERIALS AND METHODS

### 2.1 1. Animal and treatment

All experimental procedures were conducted based on the Guideline for the Care and Use of Laboratory Animals (NIH), and the animal experiment protocol approved by our animal ethics committee. In this study, a total of 32 Wistar male rats weighing 310‐370 g were purchased from our animal centre and housed in an SPF animal facility. During the experiments, the rats were given an ordinary pellet diet and unlimited access to purified drinking water. The animal room was set to a temperature of 23 ± 1 degrees Celsius, and the rats were exposed to a 12 h/12 h light/dark cycle. According to a previous published study,^15^ a total of four rat groups were established in this study by simulating the airway responsiveness and mast cell activation: (1) a non‐DM group (N = 8, rats treated with PBS only); (2) a non‐DM+INDUCTION group (N = 8, rats first subject to active sensitization (as shown below) and then subsequently exposed to 100 mg/mL of ovalbumin (OA) for induction); (3) a DM+INDUCTION group (N = 8, rats first treated with 42 mg/kg of alloxan monohydrate via iv injection to trigger DM. Then, the rats were subject to active sensitization and subsequently exposed to 100 mg/mL of OA for induction); and (4) a DM+INDUCTION +INSULIN group (N = 8, rats first treated with 42 mg/kg of alloxan monohydrate via iv injection to trigger DM. Then, the rats were subject to active sensitization and subsequently exposed to 100 mg/mL of OA for induction. Finally, the rats were treated with insulin as shown below). For the injection of alloxan monohydrate, the alloxan monohydrate compound was purchased from Sigma Aldrich (St. Louis, MO) and then dissolved in saline prior to injection. Animals were rendered diabetic 10 days before the activation of sensitization, and experiments were performed 28 days after alloxan injection. Blood glucose levels were estimated immediately before the experiments. The rats in all groups were killed to collect corresponding tissue samples for subsequent analyses.

### 2.2 Diabetes mellitus induction

The rats were given 42 mg/kg of alloxan monohydrate (dissolved in saline) via iv injection to induce diabetes mellitus, whereas the control rats were given PBS only. To determine the successful induction of diabetes mellitus, the concentration of plasma glucose was assayed daily by using a blood glucose monitor (Eli Lilly, Sao Paulo, Brazil). The threshold value for the determination of the successful induction of diabetes mellitus was 11.2 mM/L.

### 2.3 Active sensitization

The rats were sensitized proactively against ovalbumin (OA) via s.c. injection before they were given 100 mg of OA (Sigma Aldrich, St. Louis, MO) and 8 mg of aluminium hydroxide dissolved in 0.2 mL of saline via i.p. injection. Two weeks later, a 2nd sensitization was done, followed by analyses carried out 7 days later.

### 2.4 Measurement of in vitro airway contraction induced by ovalbumin

The rats were sacrificed via abdominal aorta sectioning. Then, the main bronchi and the intrapulmonary bronchi were separated before the ring segments were immersed in 8 mL of Krebs‐Henseleit solution containing 1.2 mM of KH2PO4, 115.0 mM of NaCl, 2.5 mM of MgSO4.7, 4.6 mM of KCl, 11.0 mM of glucose, 2.5 mM of CaCl2, and 25.0 mM of NaHCO3. After the tissues were equilibrated for 60 minutes under 0.5 g of tension, the tension was changed to 1.0 g. Then, the isolated bronchial segments were exposed to 100 mg/mL of OA before the contractile reaction was evaluated by utilizing an F‐60 isometric transducer (Narco Biosystem, Houston, TX).

### 2.5 Insulin treatment

The impact of insulin was studied under 2 scenarios: (1) after the diabetic rats were given a single dosage of 4 mU/mL of insulin via s.c. injection at 4 hours prior to the separation of the intrapulmonary bronchi and main bronchi; (2) after the segmented intrapulmonary bronchi and main bronchi tissues were exposed for 10 min to 1 mU/mL of crystalline insulin (Biobras, Sao Paulo, Brazil) prior to in vitro challenge.

### 2.6 Cell culture and transfection

RBL‐2H3 cells were acquired from the Cell Center of Shanghai Institutes for Biological Sciences (Shanghai, China) and incubated in DMEM consisting of 10% foetal bovine serum (Gibco, Grand Island, NY) and suitable antibiotics. The incubation was carried out in a fully humidified incubator with 5% carbon dioxide at 37°C. To study the effects of various targets, the RBL‐2H3 cells were divided into a total of 5 groups, that is 1. NC group (RBL‐2H3 cells treated with PBS only); 2. INDUCTION+Scramble siRNA group (RBL‐2H3 cells transfected with a Scramble siRNA); 3. INDUCTION+IR siRNA group (RBL‐2H3 cells transfected with siRNA targeting IR); 4. INDUCTION+IGF1R siRNA group (RBL‐2H3 cells transfected with siRNA targeting IGF1R); and 5. INDUCTION+IR siRNA+IGF1R siRNA group (RBL‐2H3 cells transfected with both siRNAs targeting IR and IGF1R). The RBL‐2H3 cells in various groups were transfected with respective siRNAs for 2 days using Lipofectamine 2000 (Invitrogen, Carlsbad, CA). Then, the cells were collected for analyses.

### 2.7 Cell sensitization

RBL‐2H3 cells were treated with 250 ng/mL of anti‐2,4‐dinitrophenyl IgE (Sigma Aldrich, St. Louis, MO) for 24 hours before then were rinsed two times in a Tyrode's buffer containing 5 mM of KCl, 135 mM of NaCl, 1.8 mM of CaCl2, 5.6 mM of glucose, 1.0 mM of MgCl2, 1 mg/mL of bovine serum albumin and 20 mM of HEPES (pH 7.4). Then, the cells were induced for 4 hours in the presence or absence of 100 ng/mL anti‐2,4‐dinitrophenyl IgE.

### 2.8 Degranulation measurement

After 24 hours of transfection, the cultured cells were sensitized by using 250 ng/mL of anti‐2,4‐dinitrophenyl IgE, followed by 1 hour of stimulation with 100 ng/mL of anti‐2,4‐dinitrophenyl IgE. Then, the degranulation reaction was terminated by putting the cells on ice for 10 minutes. To assay the level of β‐hexosaminidase and histamine secreted from the cells, a histamine ELISA kit and a β‐hexosaminidase assay kit (Thermo Fisher Scientific, Waltham, MA) were used based on the procedures recommended by the manufacturer.

### 2.9 RNA isolation and real‐time PCR

Total RNA content was isolated from samples of cultured cells and dissected tissues by using a Trizol reagent (Invitrogen, Carlsbad, CA). Then, the reverse transcription from isolated Total RNA to cDNA was carried out by using a Bestar RT assay kit (Applied Biosystems, Foster City, CA). In the next step, real‐time PCR was done by utilizing the Agilent Stratagene Mx3000 real‐time qPCR equipment (Agilent Stratagene, San Diego, CA) along with a Bestar SYBR Green master Mix (Applied Biosystems, Foster City, CA). Finally, the relative expression of IR mRNA and IGF1R mRNA was calculated by using the 2^− ΔΔCt^ approach.

### 2.10 Western blot analysis

Protein samples extracted from cells and tissue samples using a RIPA lysis buffer (Sigma Aldrich, St. Louis, MO) were evaluated by using a bicinchoninic acid (BCA) assay kit (Pierce, Waltham, MA). Then, the protein samples were resolved by SDS‐PAGE and transferred onto a polyvinylidene difluoride (PVDF) membrane (Bio‐Rad, Hercules, CA), which was then blocked for 1 hour with 5% skim milk before it was incubated in succession with anti‐IR and anti‐IGF1R primary antibodies and proper HRP‐conjugated secondary antibodies (all antibodies were purchased from Abcam, Cambridge, MA). Finally, the protein bands were developed by using an enhanced chemiluminescence reagent and visualized.

### 2.11 ELISA

The levels of IL‐1b, TNF‐a, CINC‐1, P‐selectin and β‐hexosaminidase in collected samples were determined by using commercial ELISA assay kits (Cusabio Biotech, Houston, TX) based on the manufacturer manual.

### 2.12 Radioenzymatic assay

Release of histamine in main bronchi, intrapulmonary bronchi and bronchoalveolar lavage isolated from each rat group was measured by using a radioenzymatic assay (Thermo Fisher Scientific, Waltham, MA) based on the manual instruction.

### 2.13 Statistical analysis

Results were shown as average ± SEM. One‐way ANOVA was used to compare the results of different groups, with Tukey's test being utilized as the post hoc test. A *P* value of ≤0.05 was deemed significant. All statistical analyses were done by using SPSS 21.0 (IBM, Chicago, IL).

## 3 RESULTS

### 3.1 Effect of insulin on the airway contraction in rats

**FIGURE 1:**
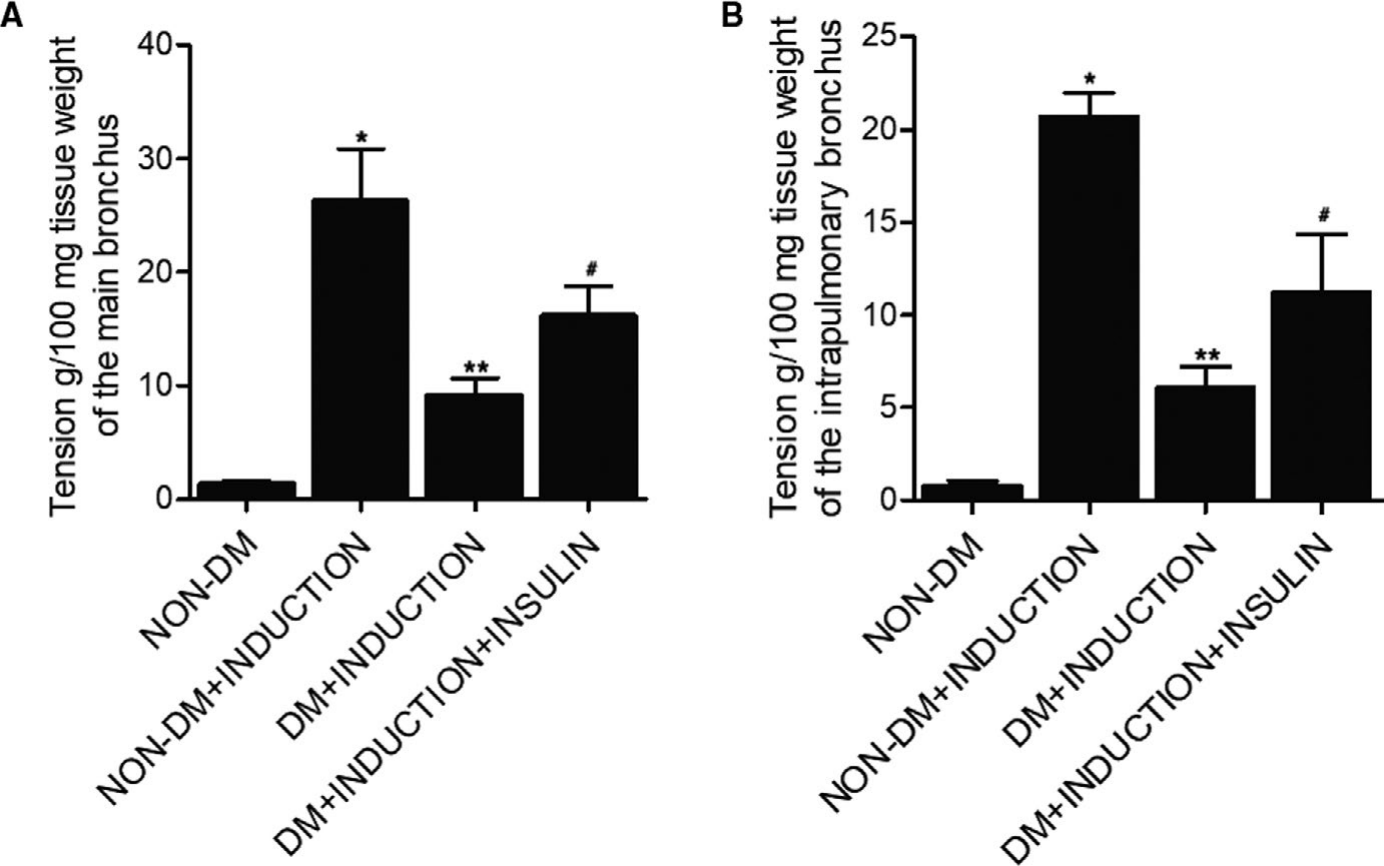
Effect of insulin on the airway contraction in rats (*P < 0.05 vs non‐DM group; **P < 0.05 vs non‐DM+INDUCTION group; #*P* < 0.05 vs DM+INDUCTION group). A, The airway contraction of main bronchi in non‐DM group, non‐DM+INDUCTION group, DM+INDUCION group and DM+INDUCTION+INSULIN group. B, The airway contraction of intrapulmonary bronchi in non‐DM group, non‐DM+INDUCTION group, DM+INDUCION group and DM+INDUCTION+INSULIN group

The airway contraction of main bronchi (Figure 1A) and intrapulmonary bronchi (Figure 1B) isolated from each rat group was measured. Accordingly, the airway contraction in DM rats showed significantly higher tension in main and intrapulmonary bronchi compared with that in the non‐DM rats, and the tension in the main and intrapulmonary bronchi isolated from DM+INDUCTION rats was lower than that of the non‐DM+INDUCTION rats. In addition, the treatment of insulin partly restored the normal level of OA‐induced airway contraction in DM rats.

### 3.2 Effect of insulin on the release of histamine in rats

**FIGURE 2:**
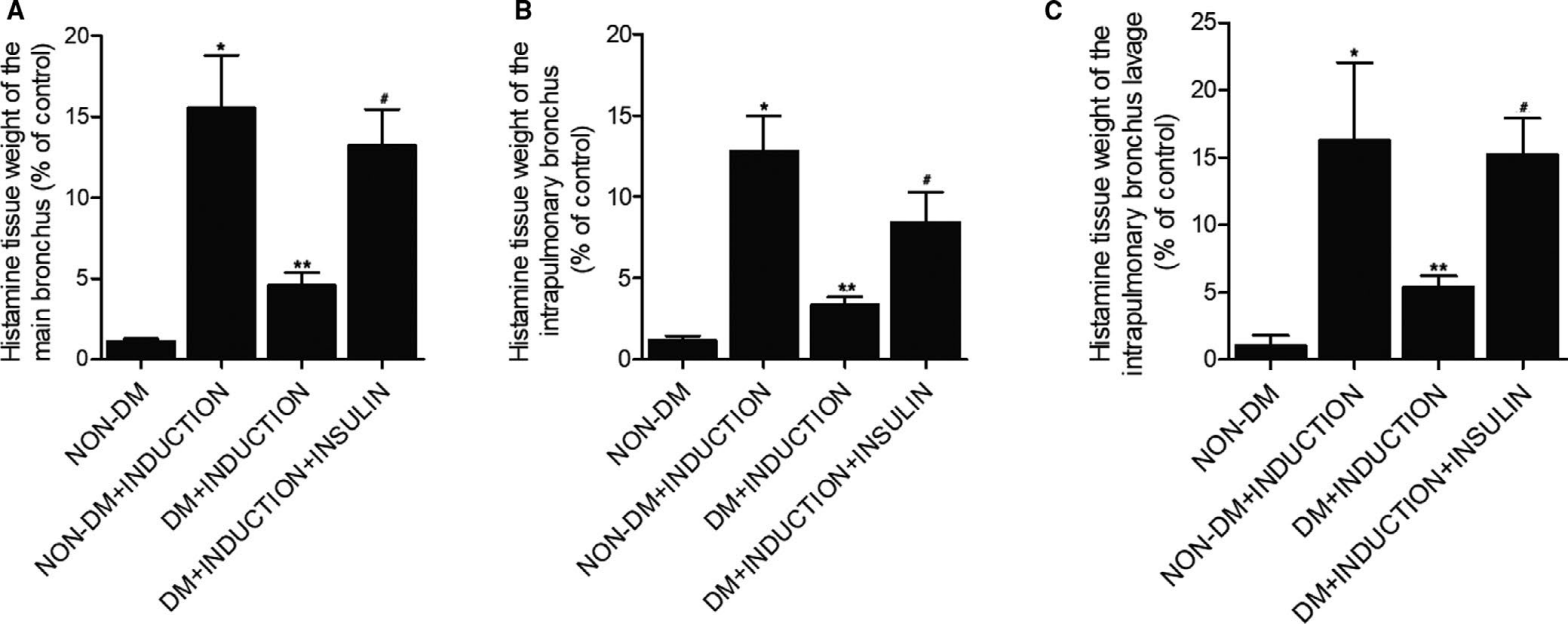
Effect of insulin on the release of histamine in rats (*P < 0.05 vs non‐DM group; **P < 0.05 vs non‐DM+INDUCTION group; #*P* < 0.05 vs DM+INDUCTION group). A, Release of histamine in main bronchi in the non‐DM group, non‐DM+INDUCTION group, DM+INDUCTION group and DM+INDUCTION+INSULIN group. B, Release of histamine in intrapulmonary bronchi in the non‐DM group, non‐DM+INDUCTION group, DM+INDUCTION group and DM+INDUCTION+INSULIN group. C, Release of histamine in bronchoalveolar lavage in the non‐DM group, non‐DM+INDUCTION group, DM+INDUCTION group and DM+INDUCTION+INSULIN group

Release of histamine in main bronchi (Figure 2A), intrapulmonary bronchi (Figure 2B) and bronchoalveolar lavage (Figure 2C) isolated from each rat group was measured using a radioenzymatic assay, which showed that the release of histamine was barely detectable in samples collected from non‐DM rats. The stimulation by OA remarkably increased the histamine release in non‐DM rats, whereas the OA‐stimulated DM rats showed substantially suppressed histamine release. Moreover, the markedly reduced release of histamine in DM+INDUCTION rats was increased by insulin.

### 3.3 Effect of insulin on the levels of IL‐1b, TNF‐a, CINC‐1 and P‐selectin in rats

**FIGURE 3:**
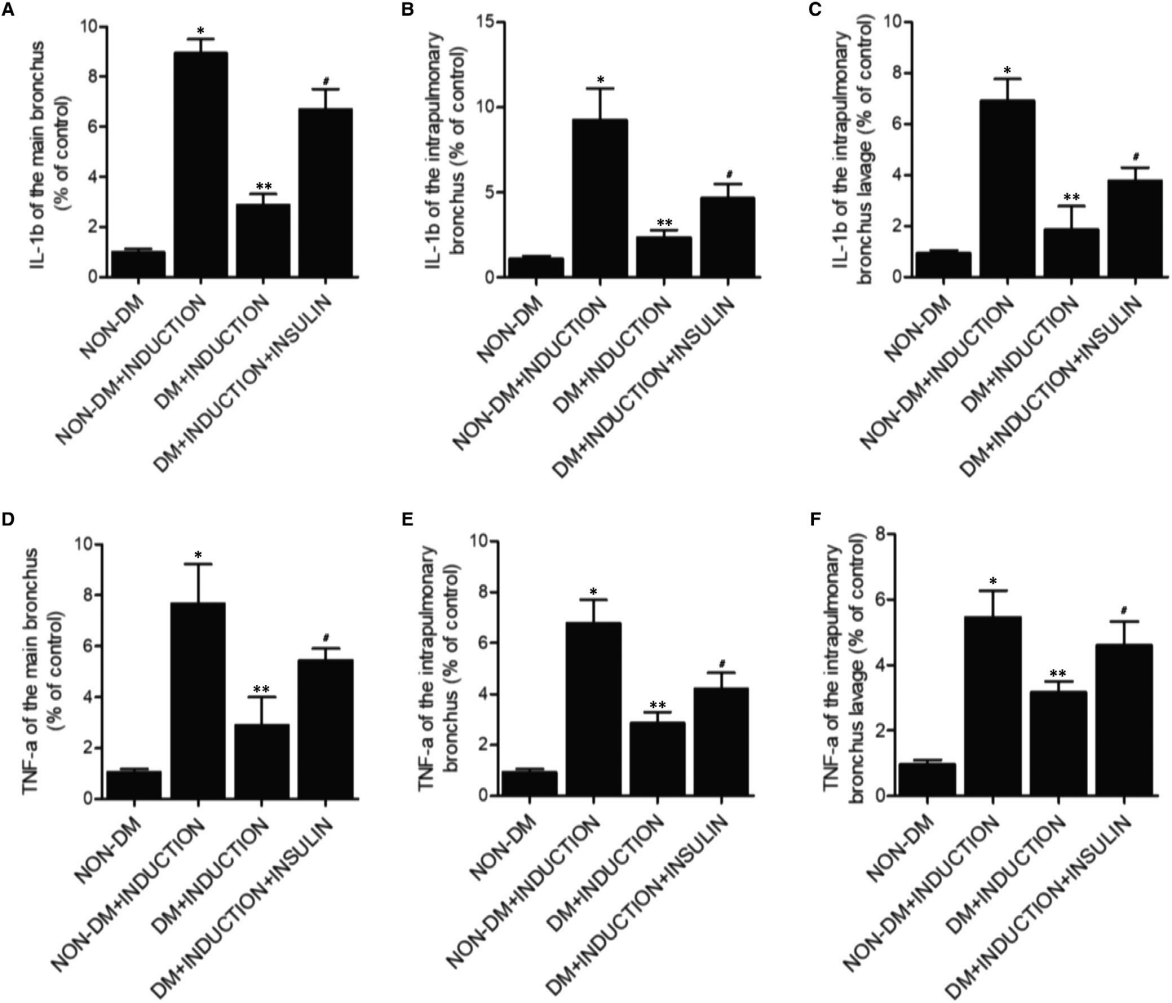
Effect of insulin on the levels of IL‐1b and TNF‐a in rats (*P < 0.05 vs non‐DM group; **P < 0.05 vs non‐DM+INDUCTION group; #*P* < 0.05 vs DM+INDUCTION group). A, Concentration of IL‐1b in main bronchi in the non‐DM group, non‐DM+INDUCTION group, DM+INDUCTION group and DM+INDUCTION+INSULIN group. B, Concentration of IL‐1b in intrapulmonary bronchi in the non‐DM group, non‐DM+INDUCTION group, DM+INDUCTION group and DM+INDUCTION+INSULIN group. C, Concentration of IL‐1b in bronchoalveolar lavage in the non‐DM group, non‐DM+INDUCTION group, DM+INDUCTION group and DM+INDUCTION+INSULIN group. D, Concentration of TNF‐a in main bronchi in the non‐DM group, non‐DM+INDUCTION group, DM+INDUCTION group and DM+INDUCTION+INSULIN group. E, Concentration of TNF‐a in intrapulmonary bronchi in the non‐DM group, non‐DM+INDUCTION group, DM+INDUCTION group and DM+INDUCTION+INSULIN group. F, Concentration of TNF‐a in bronchoalveolar lavage in the non‐DM group, non‐DM+INDUCTION group, DM+INDUCTION group and DM+INDUCTION+INSULIN group

**FIGURE 4:**
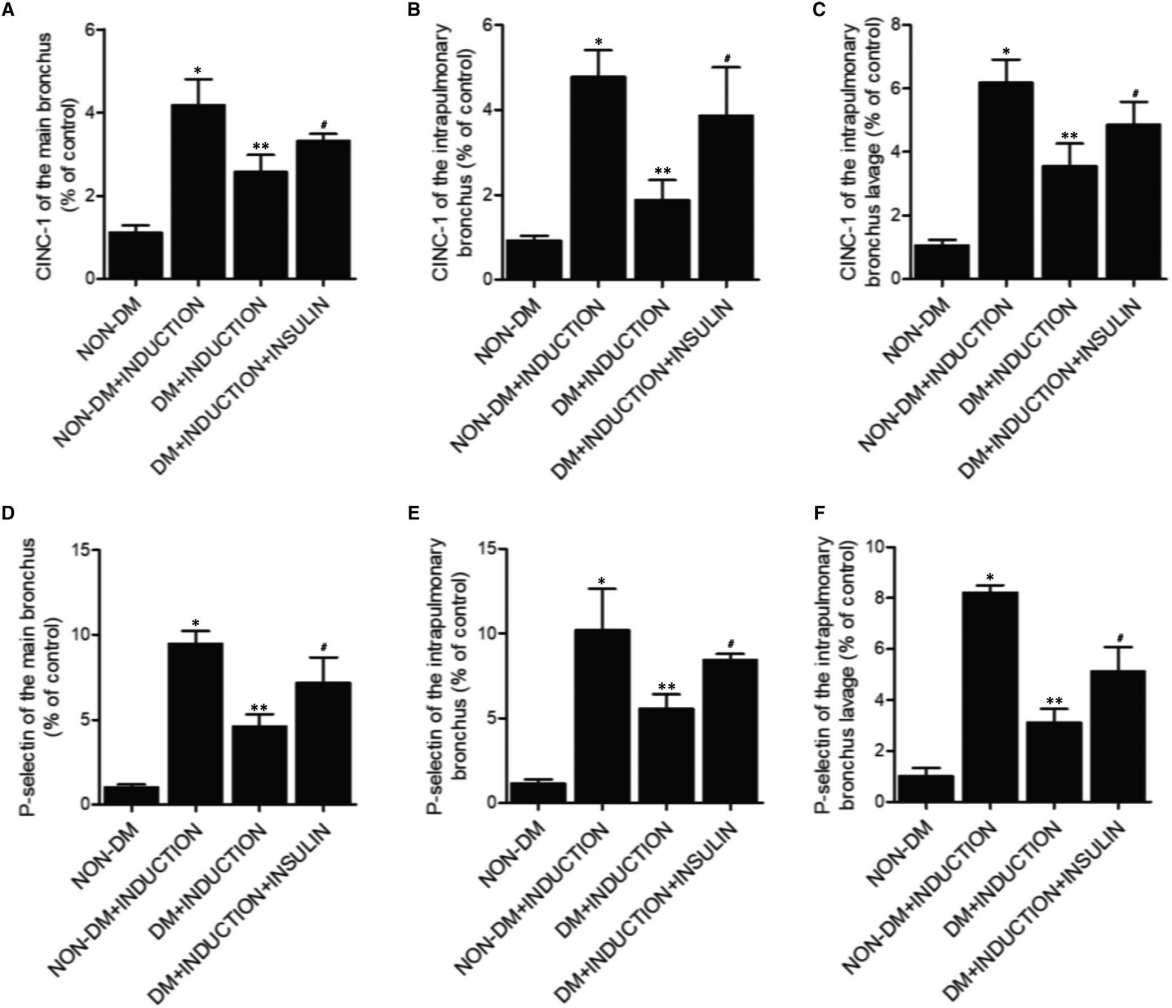
Effect of insulin on the levels of CINC‐1 and P‐selectin in rats (*P < 0.05 vs non‐DM group; **P < 0.05 vs non‐DM+INDUCTION group; #*P* < 0.05 vs DM+INDUCTION group). A, Concentration of CINC‐1 in main bronchi in the non‐DM group, non‐DM+INDUCTION group, DM+INDUCTION group and DM+INDUCTION+INSULIN group. B, Concentration of CINC‐1 in intrapulmonary bronchi in the non‐DM group, non‐DM+INDUCTION group, DM+INDUCTION group and DM+INDUCTION+INSULIN group. C, Concentration of CINC‐1 in bronchoalveolar lavage in the non‐DM group, non‐DM+INDUCTION group, DM+INDUCTION group and DM+INDUCTION+INSULIN group. D, Concentration of P‐selectin in main bronchi in the non‐DM group, non‐DM+INDUCTION group, DM+INDUCTION group and DM+INDUCTION+INSULIN group. E, Concentration of P‐selectin in intrapulmonary bronchi in the non‐DM group, non‐DM+INDUCTION group, DM+INDUCTION group and DM+INDUCTION+INSULIN group. F, Concentration of P‐selectin in bronchoalveolar lavage in the non‐DM group, non‐DM+INDUCTION group, DM+INDUCTION group and DM+INDUCTION+INSULIN group

ELISA assay was performed to measure the concentrations of IL‐1b (Figure 3A‐C), TNF‐a (Figure 3D‐E), CINC‐1 (Figure 4A‐C) and P‐selectin (Figure 4D‐E) in main bronchi (Figures 3A, 4A), intrapulmonary bronchi (Figures 3B and 4B) and bronchoalveolar lavage (Figures 3C, 4C) collected from different rat groups. Compared with the non‐DM rats, the induction of OA significantly increased the levels of IL‐1b, TNF‐a, CINC‐1 and P‐selectin. However, the induction of OA in DM rats showed a milder effect compared with the non‐DM rats, and the treatment of insulin in DM+INDUCTION rats increased the down‐regulated IL‐1b, TNF‐a, CINC‐1 and P‐selectin in DM rats.

### 3.4 The mRNA and protein expression of IR and IGF1R was inhibited in DM rats

**FIGURE 5:**
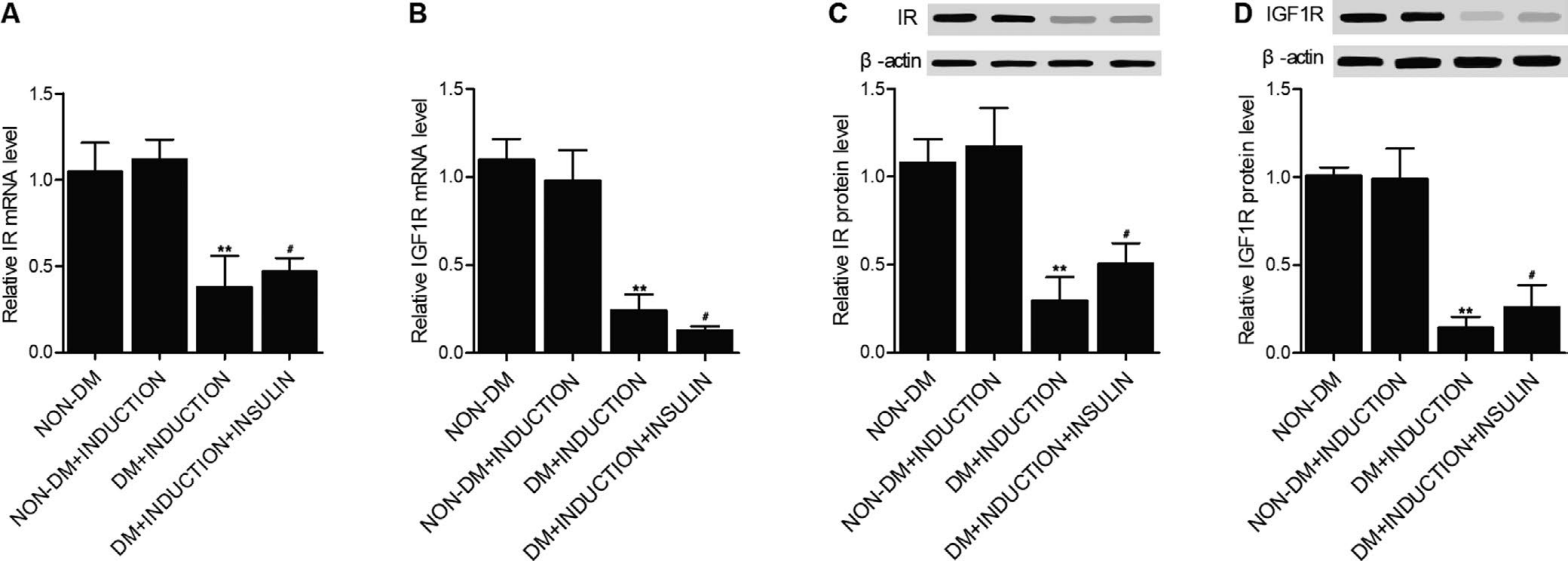
The mRNA and protein expression of IR and IGF1R was inhibited in DM rats (**P < 0.05 vs non‐DM+INDUCTION group; #*P* < 0.05 vs DM+INDUCTION group). A, The expression of IR mRNA in the non‐DM group, non‐DM+INDUCTION group, DM+INDUCTION group and DM+INDUCTION+INSULIN group. B, The expression of IGF1R mRNA in the non‐DM group, non‐DM+INDUCTION group, DM+INDUCTION group and DM+INDUCTION+INSULIN group. C, The expression of IR protein in the non‐DM group, non‐DM+INDUCTION group, DM+INDUCTION group and DM+INDUCTION+INSULIN group. D, The expression of IGF1R protein in the non‐DM group, non‐DM+INDUCTION group, DM+INDUCTION group and DM+INDUCTION+INSULIN group

The expression of IR mRNA (Figure 5A) and IGF1R mRNA (Figure 5B) was measured by real‐time PCR, and results showed that the mRNA expression of IR and IGF1R was similar between non‐DM and non‐DM+INDUCTION rats. However, in DM rats, the IR and IGF1R mRNA expression was evidently suppressed. Also, Western blot analysis showed that the expression of IR protein (Figure 5C) and IGF1R protein (Figure 5D) was both inhibited in DM rats compared with that in non‐DM rats.

### 3.5 Methylation of IR and IGF1R promoters was increased in DM rats

**FIGURE 6:**
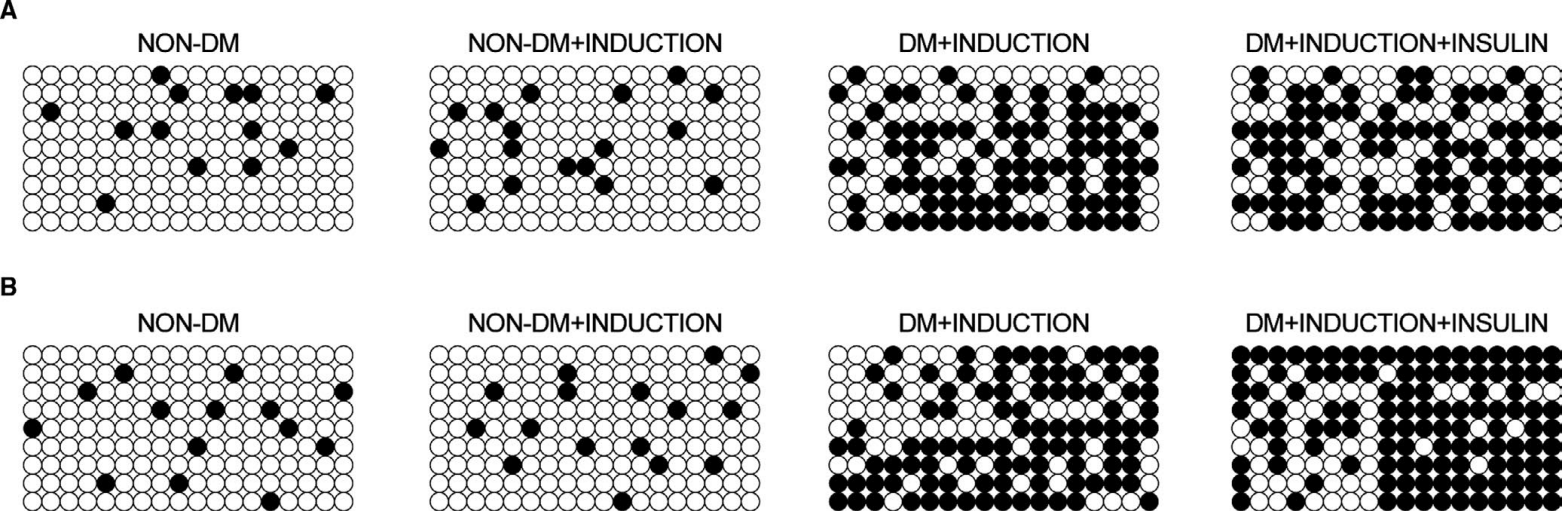
Methylation of IR and IGF1R promoters was increased in DM rats (*P < 0.05 vs NC group). A, The methylation status of IR promoter in the non‐DM group, non‐DM+INDUCTION group, DM+INDUCTION group and DM+INDUCTION+INSULIN group. B, The methylation status of IGF1R promoter in the non‐DM group, non‐DM+INDUCTION group, DM+INDUCTION group and DM+INDUCTION+INSULIN group

To further investigate the reason why IR and IGF1R were down‐regulated in DM rats, the methylation status of IR promoter (Figure 6A) and IGF1R promoter (Figure 6B) was observed by bisulphite sequencing. Accordingly, the methylation of both IR promoter and IGF1R promoter was evidently reduced in non‐DM rats compared with that in DM rats, indicating that the methylation of IR and IGF1R promoters was increased in DM rats.

**FIGURE 7:**
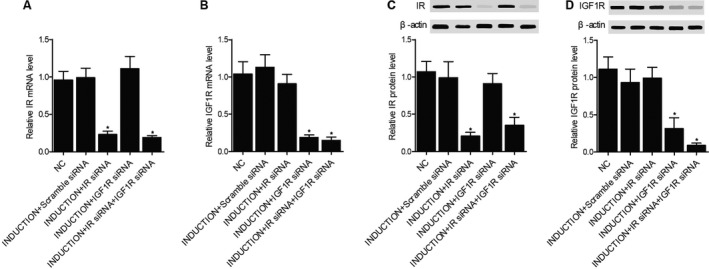
Establishment of different cell groups (*P < 0.05 vs NC group; **P < 0.05 vs induction+scramble siRNA group). A, The expression of IR mRNA in the negative control group, induction+scramble siRNA group, induction+IR siRNA group, induction+IGF1R siRNA group and induction+IR siRNA+IGF1R siRNA group. B, The expression of IGF1R mRNA in the negative control group, induction+scramble siRNA group, induction+IR siRNA group, induction+IGF1R siRNA group and induction+IR siRNA+IGF1R siRNA group. C, The expression of IR protein in the negative control group, induction+scramble siRNA group, induction+IR siRNA group, induction+IGF1R siRNA group and induction+IR siRNA+IGF1R siRNA group. D, The expression of IGF1R protein in the negative control group, induction+scramble siRNA group, induction+IR siRNA group, induction+IGF1R siRNA group and induction+IR siRNA+IGF1R siRNA group

In our study, RBL‐2H3 cells were divided into a negative control group, an induction+scramble siRNA group, an induction+IR siRNA group, an induction+IGF1R siRNA group and an induction+IR siRNA+IGF1R siRNA group. The expression of IR mRNA (Figure 7A) and protein (Figure 7C) and IGF1R mRNA (Figure 7B) and protein (Figure 7D) was compared among different cell groups. The IR mRNA and protein expression was similar among the negative control, induction+scramble siRNA and induction+IGF1R siRNA groups, whereas both the induction+IR siRNA and induction+IR siRNA+IGF1R siRNA groups showed evidently decreased expression of IR mRNA and protein. Also, the IGF1R mRNA and protein expression was similar among the negative control, induction+scramble siRNA and induction+IR siRNA groups, whereas the induction+IGF1R siRNA and induction+IR siRNA+IGF1R siRNA groups showed down‐regulated IGF1R mRNA and protein expression. Therefore, the transfection of IR siRNA and IGF1R siRNA was successful.

### 3.6 Knockdown of IR and IGF1R up‐regulated the expression of β‐hexosaminidase and histamine

**FIGURE 8:**
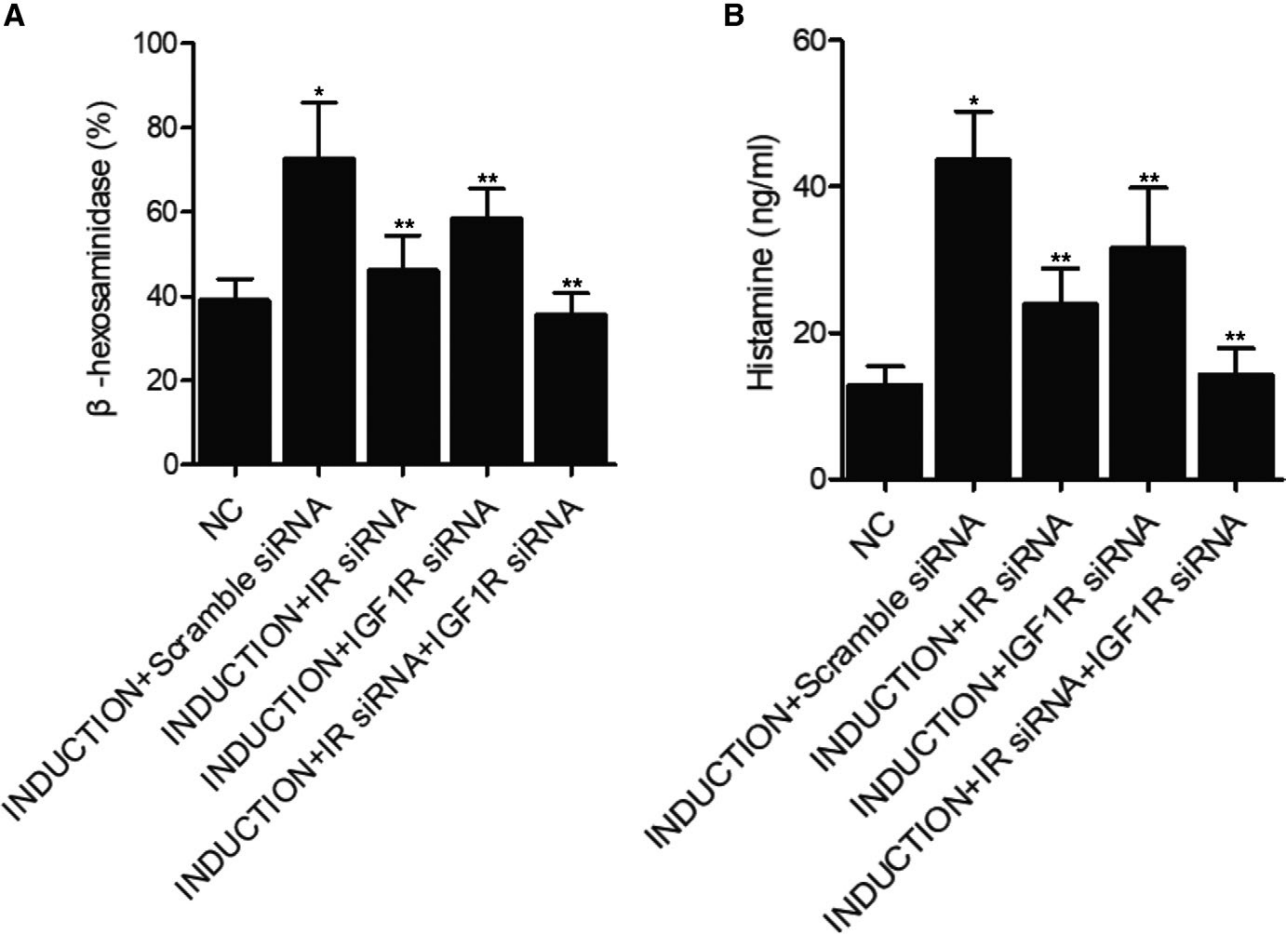
Knockdown of IR and IGF1R up‐regulated the expression of β‐hexosaminidase and histamine (*P < 0.05 vs NC group; **P < 0.05 vs induction+scramble siRNA group). A, The level of β‐hexosaminidase in the negative control group, induction+scramble siRNA group, induction+IR siRNA group, induction+IGF1R siRNA group and induction+IR siRNA+IGF1R siRNA group. B, The concentration of histamine in the negative control group, induction+scramble siRNA group, induction+IR siRNA group, induction+IGF1R siRNA group and induction+IR siRNA+IGF1R siRNA group

The level of β‐hexosaminidase (Figure 8A), which indicated the degranulation of mast cells, was compared among different cell groups. The level of β‐hexosaminidase was most evidently up‐regulated in the induction+scramble siRNA group, whereas the knockdown of IR and IGF1R both suppressed the level of β‐hexosaminidase, indicating that the knockdown of IR or IGF1R could rescue the degranulation of mast cells. Also, similar results were obtained in terms of histamine concentration (Figure 8B) in different cell groups, indicating that the down‐regulation of IR or IGF1R up‐regulated the level of histamine.

## 4 DISCUSSION

In this study, we found that the airway contraction in non‐DM rats showed significantly higher tension in main and intrapulmonary bronchi compared with that in DM rats. In addition, we found that the release of histamine was barely detectable in main and intrapulmonary bronchi or bronchoalveolar lavage of non‐DM rats. The stimulation of OA remarkably increased the histamine release in non‐DM rats, whereas the OA‐stimulated DM rats showed substantially suppressed histamine release. Moreover, the markedly reduced release of histamine in DM+INDUCTION rats was rescued in DM+INDUCTION+INSULIN rats. Previous research showed a strong correlation between the onset of type 1 diabetes mellitus and asthma in both Europe and the United States. There was actually a trend of a higher incidence of both type 1 diabetes mellitus and asthma in rich nations. The occurrence of diabetes mellitus also appeared to become much more frequent in Europe for a specific sign of asthma.^16^

The mast cells in human lungs are linked to the activity of airway smooth muscles along with the problem of airway inflammation and the activation of β2 adrenoceptor.^17^, ^18^, ^19^ Lately, it was suggested that mast cells may play a role in the advancement of type I diabetes through regulating the actions of T regulatory cells.^20^

It was revealed that reduced expression of IR seemed to be significantly associated with the aetiopathophysiology of GDM. Following changes to the methylation of IR promoter, the transcription of relevant genes was also affected. The patterns of DNA methylation in CB of GDM were likewise affected.^10^ Similarly, novel regulators of IR might likewise offer advantages in enhancing sensitivity to insulin and reducing the level of glucose in the blood of T2DM patients. Faced by adverse effects of thiazolidinediones, hypoglycaemic medications with novel targets are very attractive.^21^, ^22^ It was also confirmed that BBR activity of InsR in human was linked to its effect on lowering glucose levels.^23^

IR has actually been linked to various non‐metabolic illnesses including asthma and cancer.^24^, ^25^, ^26^ Although the link between hyperglycaemia and insulin deficiency has been examined in detail in diabetic patients, the impairing impacts of the excess level of insulin are not well acknowledged. Essentially, with the tissue‐ and cell‐specific heterogeneity in the signalling pathways of insulin, the impacts of insulin on other tissues might not be applicable to the lungs. Some scholars reviewed both the extent and pattern of epigenetic methylation in IGF1R and InsR promoters in db/db mice, which showed that the level of methylation in IGF1R promoter was elevated in the skeletal muscles of male db/db mice. However, the level of methylation in InsR promoter showed no difference between male db/db mice and control mice. The levels of IGF1R and InsR expression were also examined in muscle specimens, and the results showed an excellent correlation between the expression of IGF1R or InsR and the level of their promoter methylation.^27^

One analysis utilized a polygenic animal model of diet induced obesity to examine the function of insulin in respiratory tract hyper‐responsiveness related to obesity. It was discovered that in reaction to the electrical stimulation of vagus nerves, the level of bronchoconstriction was substantially enhanced in obese‐prone rats given a high fat diet, revealing that the hyper‐responsiveness of respiratory tract is not dependent on the diet.^28^ In this study, we found that the mRNA and protein expression of IR and IGF1R was similar between non‐DM rats and non‐DM+INDUCTION rats, whereas the IR and IGF1R mRNA expression was evidently suppressed in DM rats. The methylation of both IR and IGF1R promoters was evidently lower in non‐DM rats compared with that in DM rats, indicating that the methylation of IR and IGF1R promoters was increased in DM rats. Furthermore, we found that as compared with that in non‐DM rats, the induction of OA significantly increased the levels of IL‐1b, TNF‐a, CINC‐1 and P‐selectin. However, the induction of OA in DM rats did not exhibit an effect as evident as that in non‐DM rats, and the treatment of DM+INDUCTION rats by insulin partly restored the normal levels of IL‐1b, TNF‐a, CINC‐1 and P‐selectin down‐regulated in DM rats.

A research on endobronchial biopsy samples collected from asthma patients has actually revealed that the mRNA expression of IGF‐I was dramatically raised due to subepithelial fibrosis.^29^ These results indicated that IGF‐I might play a role of growth factor during the inflammation and hyper‐responsiveness of respiratory tract. In addition, the administration of IGF‐I neutralizing antibodies to OVA‐challenged mice reduced the level of respiratory tract inflammation and airway resistance, while increasing the wall thickening of the respiratory tract, suggesting that the inhibition of the IGF‐I signalling pathway might be used as an encouraging approach to treat asthma.^30^, ^31^ The IGF‐1 growth factor has been shown to be secreted from several types of inflammatory cells and the smooth muscle of the airway into the respiratory tract.^32^

In this study, it was shown that IR mRNA and protein expression was similar among the negative control, induction+scramble siRNA and induction+IGF1R siRNA groups, whereas both the induction+IR siRNA and induction+IR siRNA+IGF1R siRNA groups showed evidently decreased mRNA and protein expression of IR. Also, the mRNA and protein expression of IGF1R was similar among the negative control, induction+scramble siRNA and induction+IR siRNA groups, and the induction+IGF1R siRNA and induction+IR siRNA+IGF1R siRNA groups showed down‐regulated mRNA and protein expression of IGF1R. The levels of β‐hexosaminidase and histamine were most evidently up‐regulated in the induction+scramble siRNA group, whereas the knockdown of IR and IGF1R both suppressed the level of β‐hexosaminidase.

However, there are limitations of this study. Apart from the animal study, large‐scale population‐based epidemiology study is preferred. Meanwhile, as this research is focused on the study of mast cell activation, DM could affect asthma through multiple signalling pathways initiated by multiple genes. Therefore, comprehensive study involving multiple signalling pathways is warranted in the future. The results of this present study provided us a new understanding of the pathogenesis of asthma as well as the regulation of mast cell activation and also indicated a novel way to prevent or treat the asthma by inducing hypermethylation of IR and IGFR.

## 5 CONCLUSION

In this study, we demonstrated that DM‐induced hypermethylation suppressed IR and IGF1R expression, which in turn inhibited the progression of diabetes by suppressing mast cell activation and airway responsiveness. Also, insulin treatment could partly restore the normal airway responsiveness to OA in DM rats.

## CONFLICT OF INTEREST

None.

## AUTHOR CONTRIBUTIONS

Dan Fu: Conceptualization (equal); Data curation (equal); Methodology (equal); Resources (equal). Hailu Zhao: Data curation (equal); Resources (equal). Liang He: Conceptualization (equal); Data curation (equal); Funding acquisition (equal); Investigation (equal); Methodology (equal); Resources (equal); Supervision (equal); Validation (equal); Writing‐original draft (equal). Huafeng Feng: Data curation (equal); Investigation (equal); Methodology (equal). DF and LH planned the study, collected and analysed the data, and finished the manuscript. DF, HFF, and HLZ collected and analysed the data. LH reviewed the literature and the final manuscript.

## Data Availability

The datasets used and/or analysed in the current study are available from the corresponding author upon reasonable request.
